# Modified Natural Diatomite with Various Additives and Its Environmental Potential

**DOI:** 10.3390/ma16124494

**Published:** 2023-06-20

**Authors:** Krzysztof Gondek, Piotr Micek, Agnieszka Baran, Tomasz Bajda, Jerzy Kowal, Marcin Lis, Anna Wyrobisz-Papiewska, Dorota Wojtysiak, Krzysztof Smoroń

**Affiliations:** 1 Department of Agricultural and Environmental Chemistry, University of Agriculture in Krakow, Al. Mickiewicza 21, 31-120 Krakow, Poland; krzysztof.gondek@urk.edu.pl; 2 Department of Nutrition, Animal Biotechnology and Fisheries, University of Agriculture in Kraków, Al. Mickiewicza 24/28, 30-059 Krakow, Poland; piotr.micek@urk.edu.pl; 3 Department of Mineralogy, Petrography and Geochemistry, AGH University of Science and Technology, Al. Mickiewicza 30, 30-059 Krakow, Poland; bajda@agh.edu.pl; 4 Department of Zoology and Animal Welfare, University of Agriculture in Krakow, Al. Mickiewicza 24/28, 30-059 Krakow, Poland; jerzy.kowal@urk.edu.pl (J.K.); marcin.lis@urk.edu.pl (M.L.); anna.wyrobisz-papiewska@urk.edu.pl (A.W.-P.); 5 Department of Genetics, Animal Breeding and Ethology, University of Agriculture in Kraków, Al. Mickiewicza 24/28, 30-059 Krakow, Poland; dorota.wojtysiak@urk.edu.pl; 6 Specialized Mining Company “Górtech” Sp. z o.o., Ul. Galicyjska 1/43b, 31-586 Krakow, Poland; k.smoron@diato.pl

**Keywords:** diatomite, immobilisation, additives, sorption properties, ecotoxicity

## Abstract

Diatomite has recently been the subject of intensive scientific research aimed at its extensive use in industry, breeding and agriculture. The only active diatomite mine is in Jawornik Ruski, in the Podkarpacie region of Poland. Chemical pollution in the environment, including that from heavy metals, poses a threat to living organisms. Reducing the mobility of heavy metals in the environment through the use of diatomite (DT) has recently gained much interest. More effective immobilisation of heavy metals in the environment with DT, mainly through the modification of its physical and chemical properties by various methods, should be applied. The aim of this research was to develop a simple and inexpensive material showing more favourable chemical and physical properties compared with unenriched DT in terms of metal immobilisation. Diatomite (DT), after calcination, was used in the study, considering three grain fractions, i.e., 0–1 mm (DT1); 0–0.5 mm (DT2) and 5–100 µm (DT3). Biochar (BC), dolomite (DL) and bentonite (BN) were used as additives. The proportion of DTs in the mixtures amounted to 75%, and of the additive, 25%. The use of unenriched DTs after calcination poses the risk of releasing heavy metals into the environment. Enrichment of the DTs with BC and DL resulted in a reduction or absence of Cd, Zn, Pb and Ni in aqueous extracts. It was found that for the specific surface area values obtained, the additive used for the DTs was of crucial importance. The reduction in DT toxicity has been proven under the influence of various additives. The mixtures of the DTs with DL and BN had the least toxicity. The obtained results have economic importance, as the production of the best quality sorbents from locally available raw materials reduces transport costs and thus the environmental impact. In addition, the production of highly efficient sorbents reduces the consumption of critical raw materials. It is estimated that the savings from producing sorbents with the parameters described in the article can be significant in comparison with popular competitive materials of other origins.

## 1. Introduction

The problem of a deteriorating environment has recently taken on a global character [1]. The threat the human population will have to face in the near future will be that of maintaining, and subsequently restoring, the lost properties of the environment, which is an extremely difficult task. The extent of adverse environmental change is very profound and affects both living organisms and nonliving elements of the environment. One of the most dangerous effects of increasing anthropogenic impact is chemical pollution, which threatens all forms of life in the environment. Considering the level of risk, heavy metals, especially their mobile forms, pose the greatest threat [1,2]. Some heavy metals in the environment show significant reactivity even at low levels [3]. This results in the risk of their translocation into food chains and, consequently, the risk of poisoning living organisms.

Limiting the toxicity of heavy metals in the environment through the use of various organic and inorganic materials has recently gained much interest [1,3,4,5,6,7]. Despite considerable knowledge in the use of different types of materials, the most environmentally friendly solutions are still being sought by explicitly promoting the use of substances and minerals of natural origin. In addition, the effectiveness of the substance used is an important element in determining the success of immobilisation processes or the purification of the environment of active forms of heavy metals.

Diatomites are rocks formed by the transformation of diatom shells. These materials stand out for their interesting properties and many applications [1,3,8,9]. In order to improve the properties of diatomite, expensive chemical and physical functionalization methods have been developed. Chemical modification of diatomite surfaces by functionalization using coating reagents such as amino- and mercapto-silanes improves their binding capacity for certain target compounds. This type of functionalization has been used for drug carriers, optical sensors and adsorbents for application in water treatment, ion exchange and filtration. Thermal functionalization of diatomites by calcination improves the physical properties of diatomites, mainly their compressive strength. Diatomites calcinated at temperatures up to 1473 K have significant compressive strength (up to 900 kg/cm^2^).

The use of diatomites is quite common. They are used in various industries: construction, engineering (for the removal of coolants and lubricants), refractory materials and chemical industries, among others. The great importance of diatomites is in their use in agriculture, animal husbandry and in broadly understood environmental protection as an effective material for water and wastewater treatment or immobilisation of heavy metals in soil [1,4,9,10,11,12]. According to Aksakal et al. [10], the use of diatomite significantly increases the stability of soil aggregates and the moisture and field capacity of sandy soil. Ye et al. [4] believe that the application of chemically modified diatomite to chemically contaminated soil increases immobilisation of toxic heavy metals and shows potential to improve soil microbial activity.

In situ immobilisation of heavy metals is now one of the simple, effective and widely used methods for remediating these elements in the environment. It involves the introduction of various materials into soil contaminated with heavy metals in order to alter their availability by changing the physical and chemical properties of the soils. This consequently leads to the reduced mobility and bioavailability of heavy metals and, in turn, reduces the environmental risk of dispersion of these elements [13,14,15]. Materials such as zeolite, bentonite and sepiolite are widely used to reduce the in situ mobility of heavy metals and to remediate soils contaminated with these elements. This is supported by the large specific surface area of these minerals, low price, stable chemical properties and environmental friendliness [16]. Various scientific studies have shown that there are large differences in structure between the modified material used to reduce the mobility of heavy metals and the natural material. Modified or enriched with an additive, the natural material may have a higher sorption capacity and consequently a high heavy metal adsorption efficiency [12,13,14,15,16].

Currently, the discussion on the phyto-management of heavy metals in the environment using diatomite is mainly focused on improving the immobilisation efficiency of this mineral against heavy metals. Due to the situation regarding the presence of complex contaminants in the environment (multi-elemental contamination), there is little research on the effective modification of diatomite characterised by multi-element inactivation capabilities. It should also noted that diatomite, as a sedimentary rock, contains admixtures of impurities, including heavy metals. The existence of a risk of heavy metals being desorbed into the environment from the utilised diatomite may pose an efficiency problem in reducing the mobility of these elements. From an economic point of view, expensive chemical methods of diatomite purification clearly make remediation treatments more costly, so the search for alternative, cheaper and simpler methods to improve the properties of diatomite has begun.

The aim of this research was to develop a simple and inexpensive material (based on locally available raw materials) showing improved chemical, physical and ecotoxicological properties compared to diatomite alone. It was hypothesised that the enrichment of different grain fractions of DT with mineral or organic additives would improve their chemical and physical properties in terms of heavy metal immobilisation and favourably influence the ecotoxicological effect of the developed mixtures.

## 2. Materials and Methods

### 2.1. Selection of Diatomite

The test material was diatomite supplied by the “Górtech” mining company. Diatomite was extracted from a deposit located in Bircza, Poland (49°43′16.9″ N; 22°18′25.4″ E). Diatomite was subjected to a calcination process. In the procedure, a sample of 0.5 kg of diatomite was placed in a chamber furnace, and the temperature during the process was 750 °C. The sample was calcinated for 0.5 h. Different grain fractions of diatomite were included in the study, i.e., 0–1 mm (DT1); 0–0.5 mm (DT2) and 5–100 µm (DT3).

### 2.2. Materials Used as Additives

Three materials, biochar (BC), dolomite (DL) and bentonite (BN), were used as additives. Biochar (BC) was produced from coniferous biomass residues by means of pyrolysis at 500 °C; manufacturer: CarbonTeam (Krakow, Poland). The material was characterised by a relatively low ash content (9.29%), alkaline reaction (pH = 7.74) and the highest specific surface area of all the additives tested (S_BET_ = 185.6 m^2^/g). Dolomite (DL) was sourced from the PPUH ‘Dolomit’ Kopalnia Ząbkowice S.A. mine. Dolomite evidenced an alkaline pH and significantly higher ash content than biochar (96.88%). In addition, DL had the smallest specific surface area (S_BET_ = 3.0 m^2^/g) of the materials selected for testing. Commercially available calcium bentonite (BN) was also used in the experiment. The ash content of the BN used was 88.6% and the S_BET_ value was 36.2 m^2^/g. Selected properties of the diatomites (DTs) and BC, DL and BN are shown in Table 1 and Table 2. The combination method of diatomite and mineral and organic additives used in the experiment is shown in Table 3.

### 2.3. Procedure for Preparing Test Mixtures

The procedure for preparing test mixtures involved drying the DTs and mineral and organic materials at 60 °C for 24 h. Mineral and organic additives (BC, DL, BN) were ground in a laboratory grinder (0–0.2 mm). The materials thus prepared were mixed according to the scheme in Table 3.

The prepared mixtures were placed in a rotary mixer and stirred for 24 h (36 RPM). The materials obtained were subjected to chemical, physical and ecotoxicological analyses. The comparison materials consisted of DTs without additives (DT1, DT2 and DT3).

### 2.4. Chemical Analyses in Designed Materials

In the DTs without additives, as well as in the developed mixtures, pH was determined potentiometrically, and electrical conductivity (EC) was determined conductometrically in a suspension of material and redistilled water at a material:water ratio of 1:2.5. The total content of heavy metals was determined after microwave digestion of the materials in a mixture of mineral acids, HNO_3_ and HCl, maintaining a v/v ratio of 9:3. Digestion was carried out in Teflon vessels for 50 min at 195 °C, with an energy flux of 700 W. The content of selected heavy metals in the resulting solutions was determined by ICP-OES (PerkinElmer 9100DV apparatus, Shelton, CT, USA). The ash content was determined after the sample had been calcinated in a chamber oven for 8 h at 550 °C. The content of extractable forms of heavy metals from the DTs and mixtures was determined after a 24 h extraction of the sample with redistilled water (sample:extractant ratio of 1:10). The heavy metal content of the extracts obtained was determined by ICP-OES (PerkinElmer 9100DV apparatus) [17]. Immobilisation of Zn, Pb, Cd, Cu and Ni in mixtures was calculated using the following equation [18]:

(1) $$\mathrm{Immobilised}\;\mathrm{metal}\;\left(\%\right)=\frac{\left(\mathrm{water}\;\mathrm{metal}\;\mathrm{for}\;\mathrm{the}\;\mathrm{control}-\mathrm{water}\;\mathrm{metal}\;\mathrm{for}\;\mathrm{treated}\;\mathrm{sample}\right)\times 100}{\mathrm{water}\;\mathrm{metal}\;\mathrm{for}\;\mathrm{the}\;\mathrm{control}}$$

The proportion of heavy metals extracted with water in the total content was calculated according to the formula:

(2) $$\mathrm{Share}\;\mathrm{of}\;\mathrm{total}\;\left(\%\right)=\frac{\mathrm{water}\;\mathrm{metal}\;\mathrm{for}\;\mathrm{sample}\times 100}{\mathrm{total}\;\mathrm{metal}\;\mathrm{content}\;\mathrm{for}\;\mathrm{sample}}$$

### 2.5. Physical Analyses in Designed Materials

The specific surface area (S_BET_) and porosity were determined from N_2_ gas adsorption/desorption isotherms at −196 °C using an ASAP 2020 apparatus (Micromeritics, Norcross, GA, USA). Prior to measurements, the samples were outgassed for 12 h at 105 °C. Based on the data obtained from N_2_ isotherms, the S_BET_ was calculated by applying the Brunauer–Emmett–Teller (BET) equation [19]. The total pore volume was calculated from the amount of N_2_ adsorbed at a relative vapour pressure (P/P_0_) of ~0.99. The volume of micropores was calculated by applying the Dubinin–Radushkevich method [20]. The mesopore volume was determined from the adsorption branch of the isotherms by using the BJH (Barrett–Joyner–Halenda) method [21] in the mesopore range proposed by Dubinin [20]. The macropore volume (V_mac_) was calculated using the following equation:

(3) $$V_{\mathrm{mac}}=V_{\mathrm{tot}}^{0.99}-\left(V_{\mathrm{mic}}^{\mathrm{DR}}+V_{\mathrm{mes}}^{\mathrm{BJH}}\right)$$

where:

$V_{\mathrm{mic}}^{\mathrm{DR}}$—the volume of micropores
$V_{\mathrm{mes}}^{\mathrm{BJH}}$—the volume of mesopores

### 2.6. Ecotoxicity Analyses in Designed Materials

The ecotoxicity of the DTs and mixtures with mineral and organic materials was tested using three bioassays: Phytotoxkit, Ostracodtoxkit and Microtox. The Phytotoxkit and Ostracodtoxkit [22,23] assays were used to determine the toxicity of the solid phase of the samples. Two plants, *Lepidium sativum* and *Sorghum saccharatum*, were used in the Phytotoxkit assay. After the samples had been incubated for 3 days at 25 °C, the number of germinated seeds and root growth inhibition of the test plants were measured. In the Ostracodtoxkit assay, the test organism was the crustacean *Heterocypris incongruens*. For this test, mortality and growth inhibition of H. incongruens were measured after a 6-day incubation of the samples. The Microtox assay [24] was used to determine the ecotoxicity of the liquid phase (aqueous extracts). The Microtox system uses the luminescent bacteria *Alivibrio fischeri*. Exposure of the bacteria to the sample disrupts the metabolic process and reduces the amount of light produced by the bacteria. Measuring the inhibition of bacterial luminescence in a test sample relative to a control sample is a measure of toxicity. The toxicity of the DT samples and their mixtures against *Alivibrio fischeri* bacteria was investigated by performing an 81.9% screening test, using an M 500 Analyzer (Modern Water Inc., Jerseyville, IL, USA) [24]. The aqueous extract was prepared by mixing 1 volume of sample with 10 volumes of redistilled water and shaking mechanically for 24 h. Luminescence was measured before and after a 15 min incubation of the bacterial suspension with the test sample. All tests were performed according to standard procedures [22,23,24]. Screening tests were performed by analysing undiluted samples. The toxicity of the samples was assessed on the basis of the percentage of toxic effect (PE%) estimated for the biotests performed. Samples were assigned to the respective toxicity classes: class I (PE ≤ 20%; no significant toxic effect)—non-toxic sample; class II (20% < PE ≤ 50%; significant toxic effect)—low-toxic sample; class III (50% < PE < 100%; significant toxic effect)—toxic sample; class IV (PE = 100%)—very toxic sample [25].

### 2.7. Statistical Analysis

The experiment was carried out in a completely randomised design with three replicates. One-way analysis of variance (ANOVA) was carried out using Statistica v13.3 (TIBCO Software Inc., Palo Alto, CA, USA). The significance of differences between the means was evaluated using a Duncan test at *p* ≤ 0.05. For selected properties of the starting materials, a standard error (SE) was calculated for the mean value of the analysed characteristics. Pearson’s correlation was performed to identify relationships among the mobility of metals and ecotoxicity of diatomite and its mixtures as well as potential correlations among properties (pH, EC) of materials and ecotoxicity. Before starting to calculate the correlation, the data were transformed to obtain a normal distribution. Data were statistically analysed using Microsoft Excel 2016 and the Statistica v13.3 software package. The analyses were performed in triplicate.

## 3. Results

### 3.1. pH and Electrical Conductivity of DTs and Mixtures

**Figure 1:**
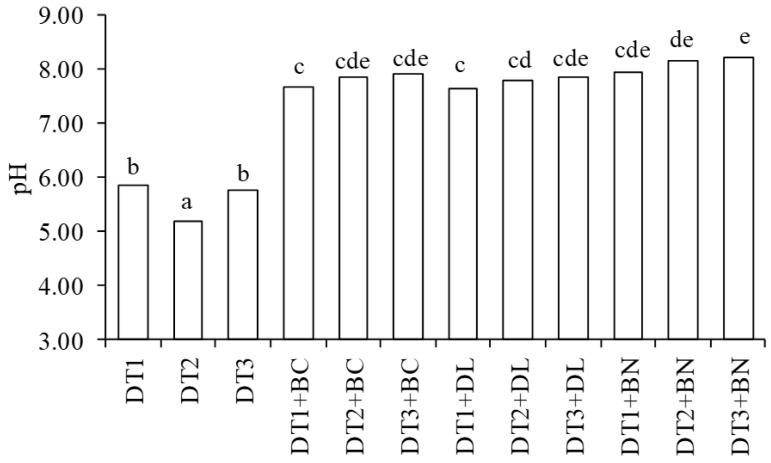
pH value of DT and mixtures of DT with mineral and organic materials. DT1—diatomite 0–1 mm, DT2—diatomite 0–0.5 mm, DT3—diatomite 5–100 µm, BC—biochar, DL—dolomite, BN—bentonite. The different letters indicate a significant difference at *p* ≤ 0.05 according to Duncan’s multiple range tests.

Compared to diatomites without additives (DT1, DT2, DT3), the pH values of mixtures with BC, DL and BN were significantly higher (*p* ≤ 0.05) (Figure 1). The highest pH values, regardless of the grain diameter of the DT used, were characterised by mixtures with bentonite (DT1 + BN, DT2 + BN, DT3 + BN).

**Figure 2:**
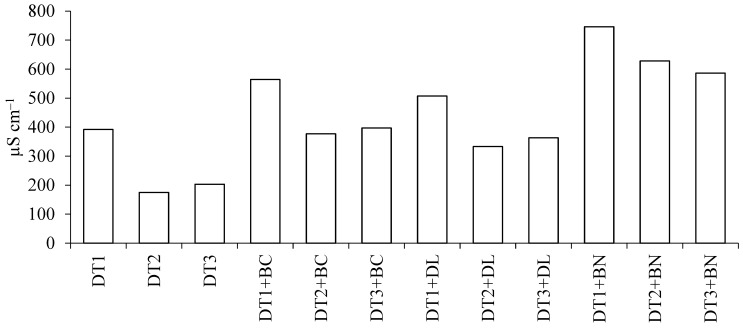
EC value of DT and mixtures of DT with mineral and organic materials. DT1—diatomite 0–1 mm, DT2—diatomite 0–0.5 mm, DT3—diatomite 5–100 µm, BC—biochar, DL—dolomite, BN—bentonite. The different letters indicate a significant difference at *p* ≤ 0.05 according to Duncan’s multiple range tests.

Diatomites without the additive, irrespective of grain diameter, were characterised by significantly (*p* ≤ 0.05) lower electrolytic conductivity (EC) values (Figure 2). The EC value increased the most in DT + BN mixtures, on average by nearly 46% compared with DT without additives.

### 3.2. Content and Mobility of Selected Heavy Metals in DTs and Mixtures

The content of the tested heavy metals, extracted with water from the DTs and mixtures of diatomites with mineral and organic materials, depended on the type of element and additive, but also on the grain diameter of the diatomite (Table 4). In general, DT3 was characterised by the lowest content of extractable heavy metals. It was found that for the batches in which DT1 was used, the content of zinc and nickel extracted with water decreased in the order (by additive) BN > DL > BC, while for lead and copper, BN > BC > DL. Cadmium extracted with water was not determined in any of the mixtures developed. For the DT2 and DT3 diatomites, the dependence of the amount of element extracted on the additive used followed the same pattern and decreased in the order BN > DL > BC.

In addition to the content of mobile forms of trace elements extracted from diatomites and mixtures of diatomites with mineral and organic materials, the contribution of these forms to the total element content is important. For cadmium (Cd), the indicator in question was not calculated due to the non-determination of extracted forms of Cd. Analysing this indicator—generally, in diatomites without additives—it was found that the proportion of extracted forms in the total content decreased with decreasing DT grain diameter (Table 5). This relationship was not confirmed for all the elements tested after mixing the DTs with different additives. The proportion of extracted forms of Zn, Pb and Cu in the total content increased in mixtures where DTs of smaller diameter were used. It should be noted that, in general, the proportion of extractable forms of Zn, Pb and Cu in mixtures with DT2 and DT3, irrespective of the additive, was at a similar level. Only the proportion of forms of extracted Ni in the total content decreased with the decrease in grain diameter of the DT used to formulate the mixture.

The immobilisation index (IM) values of the heavy metals tested differed significantly not only due to the grain diameter of the DT used to prepare the mixture, but also due to the type of additive used and the type of element (Table 5). The water extraction procedure performed for the elements tested from the DTs and their mixtures showed that, especially with the addition of BN to the DTs, there is a risk of launching heavy metals. The least amount of desorption of the heavy metals tested was recorded, irrespective of the type of element, for mixtures involving DT1. Considering the type of element, the least amount of desorption was recorded for Ni.

### 3.3. Specific Surface Area and Porosity of DTs and Mixtures

Textural parameters (specific surface area and porosity) of the DT mixtures were determined (Table 6). The S_BET_ of DTs mixed with BC is characterised by the highest values compared with the DTs + BN and DTs + DL. The higher the S_BET_ value of the additives (BC, BN, DL) to the diatomite, the higher the S_BET_ value of the resulting mixtures. BC is a predominantly microporous material of the additives tested, while DL and BN are mesoporous. Given the mesoporous nature of DT, mesopore volumes prevail over the macropore and micropore volumes in the mixtures formed.

**Figure 3:**
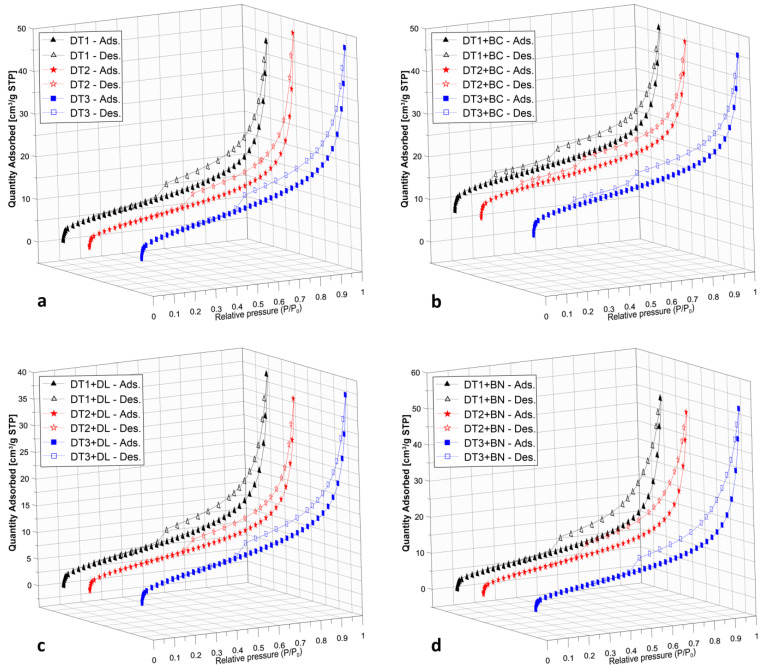
Comparison of N_2_ adsorption and desorption isotherms at −196 °C (**a**) for DT1, DT2, DT3 samples; (**b**) for DT1 + BC, DT2 + BC, DT3 + BC samples; (**c**) for DT1 + DL, DT2 + DL, DT3 + DL samples; (**d**) for DT1 + BN, DT2 + BN, DT3 + BN samples.

Based on the low-temperature adsorption and desorption of nitrogen, sorption/desorption isotherms were constructed (Figure 3). The International Union of Pure and Applied Chemistry (IUPAC) classification distinguishes I–VI types of adsorption isotherms.

Sorption/desorption isotherms of DT samples are very similar to each other (Figure 3a). The shape of the DT isotherms, independent of the particle size, corresponds to a type IV isotherm and a type H3 of the hysteresis loop. Characteristic features of the type IV isotherm are its hysteresis loop, associated with capillary condensation in mesopores, and the limiting uptake over a range of high P/P_0_. In the range of relative pressures below P/P_0_ of approximately 0.45, micropores are filled; above this value, meso- and macropores are filled. For all DT samples, hysteresis loops are visible and directly related to the capillary action of liquid nitrogen in the mesopores [26].

The DT + BC composite, consisting of microporous BC and mesoporous DT, developed a porosity dominated by DT, whose percentage in the mixture was 75% (Table 3). N_2_ adsorption/desorption experiments on DT + BC resulted in a stepwise desorption isotherm, suggesting bimodal porosity [27], while the adsorption branch shows only one step, representing the size of both the open and blocked mesopores (Figure 3b). The isotherm for the DT + DL (Figure 3c) and DT + BN (Figure 3d) samples corresponds to type IV and hysteresis loop H3, clearly indicating the mesoporous nature of the composites (Table 6). The results of the nitrogen sorption/desorption experiments on the DT samples and their compositions with BC, DL and BN did not show any significant variation in the samples depending on the DT grain fraction used. This indicates that mechanical reduction in grain size does not change the nature of the pore space. It indicates that the H3-type hysteresis loop, characteristic of samples containing DT of each grain fraction tested, defines the pore space of this material as aggregates (loose assemblages) of platelike particles forming slit-like pores.

### 3.4. Ecotoxicity of DTs and Mixtures

The study showed significant effects of diatomite and diatomite-based mixtures on the response of the test organisms (Table 7). In the Phytotoxkit assay, percentage root growth inhibition ranged from 1 to 62% (*S. saccharatum*) and −14 to 45% (*L. sativium*). Significantly (*p* ≤ 0.05), the greatest root growth inhibition for both test plants was found in the DT1 plant and the least in the DT1 + DL plant. Greater plant toxicity was observed in the DT and DT + BN sites than in the DT + BC and DT + DL sites. Furthermore, a 4-fold higher phytotoxicity of diatomite and diatomite-based mixtures was demonstrated for *S. saccharatum* than for *L. sativum*. For *L. sativum*, most sites were non-toxic, with only DT1 and DT3 + BN proving to be low-toxic. It is also noteworthy that root growth stimulation of *L. sativum* was demonstrated in the DT + BC and DT + DL sites regardless of the degree of diatomite fineness. In contrast, for *S. saccharatum*, most samples were low-toxic (DT2, DT3, DT1 + BC, DT2 + BC, DT + BN) and toxic (DT1). Subjects with DT3 + BC and DT + DL were non-toxic to this plant.

In the Ostracodtoxkit assay, inhibition of growth of *H. incongruens* ranged from 1 to 100% and mortality from 0 to 100%. Significantly (*p* ≤ 0.05), the highest mortality and growth inhibition was shown in sites with diatomite (DT) regardless of its fraction. In these sites, 100% toxicity to the crustacean was found after a 6-day incubation. High toxicity to crustaceans was also shown in mixtures with diatomite and biochar (DT + BC), particularly in the DT2 and DT3 sites. In these sites, toxicity against *H. incongruens* was 100%. The DT1 + BC mixture was low in crustacean toxicity. Significantly (*p* ≤ 0.05), the least toxicity to *H. incongruens* was shown in the diatomite (DT1) and dolomite site. It is noteworthy that all diatomite and dolomite-based mixtures (DTs + DL) were non-toxic to *H. incongruens*, with percentage toxic effects ranging from 0 to 17% (mortality) and 1 to 12% (growth inhibition), respectively (Table 7). Significant toxicity to *H. incongruens* was also found in sites with a diatomite and bentonite-based mixture (DT + BN). In these mixtures, mortality of *H. incongruens* ranged from 0 to 13% and growth inhibition from 19 to 47%.

Inhibition of luminescence of *A. fischeri* ranged from 15 to 41% (Table 7). Indeed, the highest toxicity to bacteria was observed in the DT3 diatomite site and the lowest in the diatomite–dolomite (DT2 + DL, DT3 + DL) and diatomite–biochar (DT1 + BC) mixtures. The mixed diatomite and dolomite (DTs + DL) were analysed, irrespective of fraction, and the DT1 diatomite and biochar (DT1 + BC) were non-toxic to *A. fischeri*, and luminescence inhibition in these bacteria ranged from 15 to 19%. The other mixtures were characterised by low toxicity to bacteria.

In general, a reduction in the toxicity of the DTs under the influence of various additives was found from the results obtained. According to the toxicity classification, taking into account the response of all organisms, the mixtures of diatomite and dolomite (DTs + DL) are classified as class I toxicity, which means that they are non-toxic samples that do not pose a risk to the environment. Mixtures based on diatomite and bentonite (DTs + BN) are classified as class II, which means that they are low in toxicity and also pose no risk to the environment. Class II was also awarded to the mixture based on DT1 + BC. The greatest toxicity to test organisms was shown in the DTs and DT2 + BC and DT3 + BC mixtures. These samples were classified as class IV (very toxic) (Table 7).

**Figure 4:**
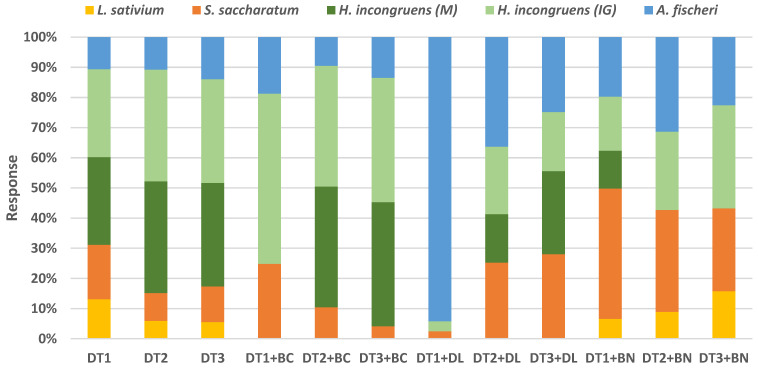
Percent share of respective biotests in test responses.

Assessing the sensitivity of the bioassays carried out showed a varied response in the test organisms. In the DTs and DTs + BC sites, the most toxic test reactions were recorded for *H. incongruens* (Figure 4). In contrast, in the DT2 + DL, DT3 + DL and DTs + BN sites, relatively many test reactions were observed for *S. saccharatum*, followed by for *A. fischeri*. The least sensitive organism proved to be *L. sativium*, irrespective of the object tested (Figure 4). Despite the different sensitivities of the organisms, the four studies showed similarity in their responses, as evidenced by significantly adding correlation coefficients (Table 8). Most significantly positive correlations were found between the luminescence inhibition of *A. fischeri* and the response of the other organisms. Significant correlations were also found between root growth inhibition of *L. sativum* and root growth inhibition of *S. saccharatum* (r = 0.74, *p* ≤ 0.05) and growth inhibition of *H. incongruens* (r = 0.40, *p* ≤ 0.05), and between mortality and growth inhibition of *H. incongruens* (r = 0.92, *p* ≤ 0.05).

Table 8 shows the correlation between the chemical properties of the tested matrices and their ecotoxicity. Most significantly positive correlations were found between the root growth inhibition of *S. saccharatum* and the content of the mobile forms of the analysed metals (except Cd), with the correlation coefficient ranging from 0.35 to 0.67. There was also a significantly positive correlation between the content of the mobile forms of Zn and Ni and the response of *L. sativium* and *A. fischeri*. Only the content of the mobile forms of Cd significantly positively correlated with the response of *H. incongruens*. Interestingly, for this organism, the most negative correlations were found between the content of the heavy metals tested and the mortality and growth inhibition of the ostracod (Table 8). Significantly positive values of the correlation coefficients indicate a relationship between the content of the element in the substrates and their toxicity to organisms, while negative values mean that the element did not increase the ecotoxicity of the sample. Of the other chemical parameters, the effect of pH and EC on the response of the test organisms was demonstrated. Correlation analysis showed negative correlations between ecotoxicity and pH (significant for *L. sativium, H. incongruens, A. fischeri*) and EC (significant for *H. incongruens*). A negative correlation relationship was observed between ecotoxicity and pH, indicating that the toxicity of the materials increased with decreasing pH values. The acid reaction of the DTs may have increased the mobility, and thus the bioavailability, of the tested heavy metals to the test organisms, as diatomite was shown to be the most toxic material (Figure 4, Table 8).

## 4. Discussion

In the study, diatomite was used as the basic component of the mixtures and was activated by means of calcination (750 °C, 0.5 h). It should be noted that the research carried out is of an applied nature; therefore, diatomite from a technological process (production line) was used. The determined differences in the pH and EC values of DT1, DT2 and DT3 resulted from the variability in the chemical composition of the raw material. The spectrum of generative and environmental processes causes diatomite rocks to be characterized by diversity in terms of some chemical indices. Diatomite is a highly variable material, containing many impurities, like terrigenous particles and inorganic oxides; it is also highly heterogeneous, as type, shape, size and fragmentation of the frustules result in unpredictable behaviour [28,29,30,31].

The results show that, irrespective of grain diameter, pure diatomites had lower pH and EC values compared with mixtures. According to Alsar et al. [28], DT contains various inorganic salts that can be released into solution as ions. It can therefore be assumed that the pH and EC values of diatomite suspensions and distilled water will depend on the quantity and quality of the desorbed ions. Studies of the kinetics of ion release from DT into solution by Alsar et al. [28] indicate that the desorption process takes 4–5 h and involves ions such as Cl^−^, SO_2_^2−^, Na^+^, Ca^2+^, Mg^2+^ and K^+^. Mixing DTs with BC, DL and BN significantly increased the pH values of the suspensions, which was due to the strongly alkaline reaction of the additives used and, therefore, the desorption of alkaline ions into solution. The mechanism of heavy metal release by mixtures of DTs with mineral and organic materials is difficult to describe due to the considerable expansion of the resulting matrix. On the one hand, the addition of different materials to DTs can increase the adsorption of many heavy metals. However, this does not exclude the occurrence of heavy metal desorption into aqueous solutions. In the experiments carried out, the addition of BC and DL to the DTs resulted in a reduction or absence of Cd, Zn, Pb and Ni in the aqueous extracts, as indicated by the IM values in addition to the determined contents. Chang et al. [32], investigating the removal of Cd(II) from aqueous solution by diatomaceous earth, showed that Ca^2+^ and Mg^2+^ in diatomaceous earth caused an ion exchange interaction that was primarily (80%) responsible for the adsorption of Cd. According to the cited authors, the remaining Cd(II) was probably trapped in the microporous structure of the diatomite. In the study by Dobor et al. [33], on a carbon-diatomaceous earth composite absorbent, the adsorption of Ni(II) and Pb(II) ions was pH-dependent, as also indicated by earlier results from [34]. Taking into account the quoted research results and the results of our own research on the developed mixtures, there is a good chance of confirming their effectiveness in immobilising heavy metals from solution at a much lower cost of sorbent production and without the use of special reagents.

According to the literature, BC is classified as a microporous material with narrow, fissured pores. Such properties are characteristic of activated carbons [26], and this is the nature of biochar. DL can be classified as a non-porous material, while BN is classified as a mesoporous material with aggregates of lamellar-like particles. The main constituent of BN is smectite-group minerals with a characteristic texture resembling a tissue layer [35]. The varied nature of the additives used in the study was reflected in the porosity of the materials developed. It was found that for S_BET_ values, the additive used for the DTs was of crucial importance. The results of nitrogen sorption/desorption experiments on samples of the DTs and their mixtures with BC, DL and BN showed no significant differences depending on the DT grain fraction used. This indicates that a mechanical reduction in DT grain size does not change the nature of the pore space.

From an ecotoxicological point of view, DT proved to be the most toxic material, regardless of the fraction. The high toxicity of DT may be due to two factors: the first factor being the reaction of this material; and the second factor being its physical properties (size and porosity) [34,35,36,37]. As shown in the study, the greatest toxicity this material showed was against *H. incongruens*. It is worth mentioning that *H. incongruens* is a very sensitive organism to the acid reaction of the substrate, and, furthermore, the oral route is the main route of exposure of this organism to toxic substances [34,35,36,37,38]. The pH values of the studied DTs ranged from 5.19 to 5.84 and were below the tolerance range indicated for *H. incongruens*. Secondly, sharp, porous and small-sized diatomite fragments can damage the digestive tract of *H. incongruens* and disrupt the functionality of the cuticle through sorption and abrasion [39]. The negative impact of diatomite on organisms is not new, as it is used as a natural pesticide in agriculture [40]. In the study by Borroso et al. [41], an increased plant root inhibition was shown in a 3-day Phytotoxkit assay in sites where diatomite was added to the soil. The observed effect of diatomite therefore seems plausible and, in addition, consistent with the literature [42]. The addition of BN and especially DL significantly reduced the toxicity of the DTs. This effect was associated with an improvement in the reaction in the substrates and a reduction in the mobility of heavy metals. The DT2 + BC and DT3 + BC sites showed the least reduction in toxicity to organisms. Biochar has a rich matrix compared to the other additives and, in addition to heavy metals, depending on its origin, it may contain various toxic organic compounds e.g., PAHs, which may be responsible for its ecotoxicity [43,44]. It is worth noting that bioassays not only allow the detection of a toxic substance, but also allow a full assessment of the effects of all agents and substances, taking into account synergism or antagonism [45].

To sum up, our research results have economic importance, as the production of the best quality sorbents from locally available raw materials reduces transport costs and thus the environmental impact. It is estimated that the savings from producing sorbents with the parameters described in the article can be significant in comparison to popular competitive materials of other origins.

## 5. Conclusions

1. The use of unenriched DTs poses the risk of releasing heavy metals into the environment.
2. Enrichment of the DTs with BC and DL resulted in a reduction or absence of Cd, Zn, Pb and Ni in aqueous extracts.
3. It was found that for S_BET_ values, the type of DT additive used was of crucial importance. The results of nitrogen sorption/desorption experiments on samples of DTs and their mixtures with BC, DL and BN showed no significant differences depending on the DT grain fraction used.
4. The reduction in DT toxicity has been proven under the influence of various additives. Taking into account the response of all organisms, DTs + DL and DTs + BN mixtures are classified as not posing an environmental risk. DT2 + BC and DT3 + BC mixtures were the most toxic to test organisms. These samples were classified as class IV (very toxic).
5. The proposed experiment confirms the legitimacy of diatomite enrichment before its environmental use.

## Figures and Tables

**Table 1:** Selected physical and chemical properties of materials.

Material *	Dry Matter g·kg^−1^	Ash g·kg^−1^	Loss on Ignition g·kg^−1^	pH H_2_O	EC ** µS·cm^−1^	BET Surface Area m^2^·g^−1^	Total Pore Volume cm^3^·g^−1^
DT1	855 ± 3	808 ± 3	192 ± 3	5.84 ± 0.05	392 ± 1	25.9 ± 0.9	0.064 ± 0.002
DT2	937 ± 4	891 ± 4	109 ± 4	5.19 ± 0.11	175 ± 2	24.6 ± 0.9	0.062 ± 0.002
DT3	932 ± 4	879 ± 2	121 ± 2	5.76 ± 0.17	203 ± 6	31.3 ± 1.1	0.067 ± 0.002
BC	952 ± 4	99 ± 0	901 ± 0	7.74 ± 0.02	330 ± 2	185.6 ± 6.6	0.088 ± 0.003
DL	942 ± 4	915 ± 3	85 ± 3	7.87 ± 0.04	107 ± 1	3.0 ± 0.1	0.009 ± 0.000
BN	949 ± 4	881 ± 3	119 ± 3	9.31 ± 0.01	842 ± 8	36.2 ± 1.3	0.115 ± 0.004

mean ± SE, * DT1—diatomite 0–1 mm, DT2—diatomite 0–0.5 mm, DT3—diatomite 5–100 µm, BC—biochar, DL—dolomite, BN—bentonite; ** EC—electrical conductivity.

**Table 2:** Total content of heavy metals in the material.

Material	Zn	Pb	Cd	Cu	Ni
	mg·kg^−1^				
DT1	40.7 ± 1.2	14.06 ± 0.08	0.34 ± 0.06	45.3 ± 0.8	21.67 ± 0.73
DT2	38.9 ± 1.1	12.57 ± 0.05	0.14 ± 0.00	44.4 ± 0.4	17.38 ± 0.58
DT3	36.8 ± 0.7	11.43 ± 0.04	0.16 ± 0.01	47.6 ± 1.9	17.52 ± 0.57
BC	30.0 ± 1.3	2.26 ± 0.40	4.95 ± 0.01	14.4 ± 2.3	18.77 ± 3.02
DL	38.8 ± 1.9	11.40 ± 0.07	1.89 ± 0.19	13.2 ± 0.7	1.61 ± 0.15
BN	28.6 ± 2.3	14.56 ± 0.05	1.06 ± 0.03	21.6 ± 2.1	1.13 ± 0.03

**Table 3:** The combination method of diatomite and mineral and organic additives.

Material	Treatment
DT1	100 g·kg^−1^
DT2	
DT3	
DT1 + BC	DT1 (75 g·kg^−1^) + BC (25 g·kg^−1^)
DT2 + BC	DT2 (75 g·kg^−1^) + BC (25 g·kg^−1^)
DT3 + BC	DT3 (75 g·kg^−1^) + BC (25 g·kg^−1^)
DT1 + DL	DT1 (75 g·kg^−1^) + DL (25 g·kg^−1^)
DT2 + DL	DT2 (75 g·kg^−1^) + DL (25 g·kg^−1^)
DT3 + DL	DT3 (75 g·kg^−1^) + DL (25 g·kg^−1^)
DT1 + BN	DT1 (75 g·kg^−1^) + BN (25 g·kg^−1^)
DT2 + BN	DT2 (75 g·kg^−1^) + BN (25 g·kg^−1^)
DT3 + BN	DT3 (75 g·kg^−1^) + BN (25 g·kg^−1^)

**Table 4:** Content of metal forms extracted with water from DT and mixtures.

Material	Cd	Zn	Pb	Cu	Ni
	mg·kg^−1^				
DT1	0.085 a	3.802 c	0.276 a	0.137 b	3.654 f
DT2	0.013 b	0.746 a	0.206 a	0.005 a	0.551 e
DT3	nd	0.271 a	0.204 a	0.018 ab	0.206 bc
DT1 + BC	nd	0.094 a	0.201 a	0.009 a	0.177 ab
DT2 + BC	nd	0.103 a	0.188 a	0.015 a	0.108 a
DT3 + BC	nd	0.107 a	0.175 a	0.025 ab	0.110 a
DT1 + DL	nd	0.164 a	0.192 a	0.007 a	0.294 c
DT2 + DL	nd	0.157 a	0.190 a	0.032 ab	0.179 ab
DT3 + DL	nd	0.165 a	0.178 a	0.036 ab	0.173 ab
DT1 + BN	nd	2.565 b	0.910 b	0.473 c	0.585 e
DT2 + BN	nd	4.222 c	1.416 c	0.792 d	0.450 d
DT3 + BN	nd	3.679 c	1.250 c	0.698 d	0.411 d

nd—not determined. The different letters within indicate a significant difference at *p* ≤ 0.05 according to Duncan’s tests.

**Table 5:** Share in the total content of Cd, Zn, Pb, Cu and Ni forms extracted with water and immobilisation coefficients (IM) in DTs and mixtures.

Material		Cd	Zn	Pb	Cu	Ni
DT1	Share of total (%)	25.20	9.35 bc	1.96 ab	0.30 b	16.85 d
	IM (%)	-	-	-	-	-
DT2	Share of total (%)	8.94	1.92	1.64	0.01	3.17 bc
	IM (%)	-	-	-	-	-
DT3	Share of total (%)	-	0.74 a	1.79 ab	0.04a	1.18 ab
	IM (%)	-	-	-	-	-
DT1 + BC	Share of total (%)	-	0.25 a	1.95 ab	0.02 a	0.77 a
	IM (%)	-	98	27	93	95
DT2 + BC	Share of total (%)	-	0.28 a	2.03 b	0.04 a	0.56 a
	IM (%)	-	86	8	−201	80
DT3 + BC	Share of total (%)	-	0.30 a	1.95 ab	0.07 a	0.57 a
	IM (%)	-	61	14	−42	47
DT1 + DL	Share of total (%)	-	0.15 a	0.60 a	0.02 a	2.07 b
	IM (%)	-	96	30	95	92
DT2 + DL	Share of total (%)	-	0.12 a	0.57 a	0.09 a	1.44 ab
	IM (%)	-	79	7	−540	67
DT3 + DL	Share of total (%)	-	0.13 a	0.53 a	0.10 a	1.38 ab
	IM (%)	-	39	13	−104	16
DT1 + BN	Share of total (%)	-	6.67 b	6.00 c	1.30 c	3.44 c
	IM (%)	-	33	−230	−246	84
DT2 + BN	Share of total (%)	-	10.71 c	10.59 d	2.26 d	3.14 bc
	IM (%)	-	−466	−589	−15814	18
DT3 + BN	Share of total (%)	-	10.64 c	10.20 d	2.00 d	3.03 bc
	IM (%)	-	−1259	−512	−3845	−100

The different letters indicate a significant difference at *p* ≤ 0.05 according to Duncan’s tests.

**Table 6:** Textural parameters of samples.

Material	S_BET_ [m^2^ g^−1^]	${V_{\mathrm{tot}}^{0.99}}^{1)}$ [cm^3^ g^−1^]	${V_{\mathrm{mic}}^{\mathrm{DR}}}^{1)}$ [cm^3^ g^−1^]	${V_{\mathrm{mez}}^{\mathrm{BJH}}}^{1)}$ [cm^3^ g^−1^]	${V_{\mathrm{mac}}}^{1)}$ [cm^3^ g^−1^]
DT1	25.9 ± 0.23	0.064	0.011	0.037	0.016
DT2	24.6 ± 0.25	0.062	0.010	0.034	0.018
DT3	31.3 ± 0.25	0.067	0.013	0.041	0.013
DT1 + BC	56.7 ± 0.83	0.067	0.022	0.035	0.010
DT2 + BC	55.6 ± 0.81	0.066	0.022	0.033	0.011
DT3 + BC	52.2 ± 0.83	0.064	0.021	0.035	0.008
DT1 + DL	19.4 ± 0.18	0.053	0.008	0.031	0.004
DT2 + DL	19.7 ± 0.22	0.046	0.008	0.027	0.011
DT3 + DL	23.5 ± 0.19	0.052	0.009	0.032	0.011
DT1 + BN	25.5 ± 0.22	0.072	0.010	0.043	0.019
DT2 + BN	27.1 ± 0.27	0.072	0.011	0.041	0.020
DT3 + BN	26.1 ± 0.23	0.076	0.011	0.045	0.020

**Table 7:** Response of test organisms and toxicity classification of samples.

Material *	*L. sativum*	*S. saccharatum*	*H. incongruens*		*A. fischeri*	Class of Toxicity
	IGR% *	IGR%	M%	IG%	IL%	
DT1	45 f	62 f	100 c	100 e	37 f	IV
DT2	16 de	25 bcd	100 c	100 e	29 d	IV
DT3	16 de	35 cde	100 c	100 e	41 g	IV
DT1 + BC	−5 ab	20 abc	0 a	44 d	15 a	II
DT2 + BC	−12 a	26 bcd	100 c	100 e	24 c	IV
DT3 + BC	−4 abc	10 a	100 c	100 e	33 de	IV
DT1 + DL	−14 a	1 a	0 a	1 a	19 b	I
DT2 + DL	−1 abc	11 a	7 ab	9 ab	15 a	I
DT3 + DL	−4 abc	17 ab	17 b	12 ab	15 a	I
DT1 + BN	7 bcd	46 e	13 b	19 bc	21 b	II
DT2 + BN	9 cde	34 bcde	0 a	26 c	32 de	II
DT3 + BN	22 e	38 de	0 a	47 d	31 de	II

* IGR%—inhibition of root growth, M%—mortality, IG%—inhibition of growth, IL%—inhibition of luminescence. The different letters indicate a significant difference at *p* ≤ 0.05 according to Duncan’s tests.

**Table 8:** Correlations between chemical properties of materials and organism responses.

Parameter	Ls IGR **	Ss IGR%	Hi M%	Hi IG%	Af IL%
Ss IGR	0.74 *				
Hi M	0.29	0.22			
Hi IG	**0.40**	0.32	**0.92**		
Af IL	**0.62**	**0.50**	**0.63**	**0.70**	
pH	**−0.54**	−0.32	**−0.66**	**−0.61**	**−0.51**
EC	−0.11	0.16	**−0.68**	**−0.55**	−0.27
Cd H_2_O	0.25	0.11	**0.58**	**0.44**	0.08
Cu H_2_O	0.31	**0.41**	**−0.46**	−0.29	0.23
Fe H_2_O	0.23	**0.35**	**−0.48**	−0.31	0.20
Mn H_2_O	**0.66**	**0.56**	0.09	0.15	**0.28**
Ni H_2_O	**0.75**	**0.65**	0.31	0.30	**0.39**
Pb H_2_O	0.26	**0.37**	**−0.47**	−0.30	0.21
Zn H_2_O	**0.66**	**0.67**	−0.19	−0.05	**0.43**
Cr H_2_O	0.19	**0.39**	**−0.44**	−0.32	0.18

* Values significant at *p* < 0.05 are shown in bold, ** Ls IGR%—inhibition of roots growth *Lepidium sativum*, Ss IGR%—inhibition of roots growth *Sorghum saccharatum*, Hi M%—mortality of *Heterocypris incongruens*, Hi IG%—inhibition of growth of *Heterocypris incongruens*, Af IL%—inhibition of luminescence of *Alivibrio fischeri*.

## Data Availability

Not applicable.
